# Inflammatory biomarker signatures in post-surgical drain fluid may detect anastomotic leaks within 48 hours of colorectal resection

**DOI:** 10.1007/s10151-023-02841-y

**Published:** 2023-07-24

**Authors:** S. M. Cuff, N. Reeves, E. Lewis, E. Jones, S. Baker, A. Karategos, R. Morris, J. Torkington, M. Eberl

**Affiliations:** 1 Division of Infection & Immunity, School of Medicine, Cardiff University, Cardiff, UK; 2 University Hospital of Wales, Cardiff & Vale University Health Board, Cardiff, UK; 3 Technical Operations, Siemens Healthineers, Llanberis, UK; 4 Systems Immunity Research Institute, Cardiff University, Cardiff, UK

**Keywords:** Colorectal, Anastomotic Leak, Biomarkers, Peritoneal fluid

## Abstract

**Background:**

The optimal treatment of colorectal cancer is surgical resection and primary anastomosis. Anastomotic leak can affect up to 20% of patients and creates significant morbidity and mortality. Current diagnosis of a leak is based on clinical suspicion and subsequent radiology. Peritoneal biomarkers have shown diagnostic utility in other conditions and could be useful in providing earlier diagnosis. This pilot study was designed to assess the practical utility of peritoneal biomarkers after abdominal surgery utilising an automated immunoassay system in routine use for quantifying cytokines.

**Methods:**

Patients undergoing an anterior resection for a rectal cancer diagnosis were recruited at University Hospital of Wales, Cardiff between June 2019 and June 2021. A peritoneal drain was placed in the proximity of the anastomosis during surgery, and peritoneal fluid was collected at days 1 to 3 post-operatively, and analysed using the Siemens IMMULITE platform for interleukin (IL)-1β, IL-6, IL-10, CXCL8, tumour necrosis factor alpha (TNFα) and C-reactive protein (CRP).

**Results:**

A total of 42 patients were recruited (22M:20F, median age 65). Anastomotic leak was detected in four patients and a further five patients had other intra-abdominal complications. The IMMULITE platform was able to provide robust and reliable results from the analysis of the peritoneal fluid. A metric based on the combination of peritoneal IL-6 and CRP levels was able to accurately diagnose three anastomotic leaks, whilst correctly classifying all negative control patients including those with other complications.

**Conclusions:**

This pilot study demonstrates that a simple immune signature in surgical drain fluid could accurately diagnose an anastomotic leak at 48 h postoperatively using instrumentation that is already widely available in hospital clinical laboratories.

## Introduction

Colorectal cancer is the third most common cancer worldwide, with the majority of patients treated with surgical resection and primary anastomosis. Between 1% and 20% of colorectal resections result in an anastomotic leak (AL) [1], which poses a significant risk for patients. As well as the immediate threat of developing faecal peritonitis and sepsis, with its attendant morbidity and mortality profile, in the long term AL is associated with a lower 5-year cancer-specific survival rates [2–7] and increased risk of cancer recurrence [2–4, 8, 9]. Hence, there is an urgent and unmet need to identify AL quickly to enable early intervention, possible anastomotic salvage and avoid long-term complications.

Current methods of diagnosing AL are usually based on serial clinical examinations and radiological imaging from days 3–5 postoperatively. These include symptoms such as pain, tachycardia, fever, oliguria, ileus, diarrhoea and leukocytosis, in combination with elevated blood levels of C-reactive protein (CRP). However, the clinical signs are non-specific and are only observed once there is a systemic response to the AL. Similarly, blood CRP can aid diagnosis, with postoperative day 3 levels of less than 172 mg/l having a negative predictive value of 97% but only a low specificity for positive diagnosis of an AL [10].

By postoperative day 3 to 5 the risk of mortality increases, alongside the necessity for an emergency operation to treat faecal peritonitis with its concomitant high risk of a permanent stoma formation. Earlier detection of an AL could change the management and treatment of these patients and, crucially, improve their postoperative outcome and quality of life.

In the present study, we examined whether peritoneal fluid drained from the surgical site can be used to diagnose AL more rapidly and accurately than current practice. By assaying peritoneal fluid in proximity to the anastomotic site, one should be able to detect the local response to colonic contamination of the peritoneum more quickly, sensitively and specifically than later systemic responses. This hypothesis was based on previous studies in which peritoneal fluid was found to respond more rapidly to the presence of AL than routinely sampled blood [11], confirming that it is more likely to be an effective medium for robust early detection. Similarly, our own research in different pathologies has shown that local immune signatures (“immune fingerprints”) at inflammatory sites are powerful predictors of acute infections, including in the peritoneal cavity of patients receiving peritoneal dialysis [12, 13], in the urine of patients presenting with suspected urinary tract infection [14], and in cerebrospinal fluid of neurosurgical patients [15]. Such disease-specific immune fingerprints combining clinical parameters with soluble and cellular biomarkers of inflammation, organ damage and/or physiology are likely to improve on the reliance on a single biomarker and increase both the sensitivity and specificity of early diagnostic tests.

In order to have immediate relevance for patient benefit, the present study was designed to assess the practical utility of biomarkers in drain fluid after abdominal surgery and test a suite of clinically approved biomarkers using the Siemens IMMULITE platform, an automated immunoassay system routinely used for quantifying cytokines in hospitals globally.

## Methods

### Ethics approval

All methods were carried out in accordance with relevant guidelines and regulations. Experimental protocols were approved by Cardiff University, and written informed consent was obtained from all subjects. Recruitment of patients was approved by the Wales Cancer Bank under reference no. 18/016 and conducted according to the principles expressed in the Declaration of Helsinki.

### Objectives

The primary objective of the study was to assess whether altered levels of peritoneal fluid biomarkers (cytokines) in response to AL could be measured on a widely available commercial immunoanalyser platform.

The secondary outcome was the analysis of peritoneal biomarker measurements to explore their sensitivity and specificity for AL diagnosis.

### Patient cohort

Between June 2019 and June 2021, all patients undergoing an elective anterior resection within the colorectal department of the University Hospital of Wales in Cardiff (UK) were screened for inclusion. Inclusion criteria were cancer diagnosis, intended primary anastomosis, age ≥ 18 years and capacity to consent. Exclusion criteria were non-cancer diagnosis, lack of capacity and a permanent end colostomy formation. Patients who had neoadjuvant chemoradiotherapy were included, and the intention to form a defunctioning ileostomy was not an exclusion criterion. Operations were performed according to surgeon preference, either laparoscopically or via a midline laparotomy. Laparoscopic procedures had extraction sites via a midline or a Pfannenstiel incision. The primary anastomosis was formed using a conventional circular stapler, the size according to surgeon choice. At the end of the operation a non-suction drain was placed in the left iliac fossa, into the pelvis. The removal of the drain was decided upon by the operating surgeon but was left in situ for at least 48 h postoperatively.

### Clinical assessments

Patient demographics were collected preoperatively. These included age, sex, body mass index, smoking status, and previous chemotherapy and/or radiotherapy. Intraoperative variables were also collected, including operation time, laparoscopic or open approach, and formation of defunctioning stoma. Postoperatively, data were collected on length of hospital stay, tumour staging (TNM) and postoperative complications. Complications were classified as AL or other complications, which included bleeding, small bowel obstruction and any other reason for return to theatre. AL was defined as clinically manifest insufficiency of the anastomosis leading to a clinical state requiring treatment (i.e. grade B/C) [16]. AL was confirmed by either computer tomography (CT) scan and/or reoperation. As such, patients were stratified into three groups for analysis: ‘uneventful recovery’, ‘anastomotic leak’ and ‘other complications’. All clinical data were incorporated into a database in which data were pseudo-anonymised by assigning each patient a study number. No personally identifiable information was included.

### Collection and storage of peritoneal fluid

Drain peritoneal fluid was collected at 8:00 am on days 1 and 2 postoperatively, as well as on day 3 if that patient still had the peritoneal drain in situ. The first sample (day 1) was collected between 14 and 20 h postoperatively. All samples were transferred for analysis in the laboratory within 30 min. The peritoneal fluid was centrifuged twice at 500×*g* for 20 min at 20 °C. The cell-free supernatant was removed, aliquoted into 2-ml cryotubes and stored at − 80 °C until further use.

### Peritoneal drain fluid analysis

Peritoneal fluid levels of interleukin (IL)-1β, IL-6, IL-8/CXCL8, IL-10, tumour necrosis factor alpha (TNFα) and CRP were measured using the corresponding IMMULITE 1000 kits on IMMULITE 1000 Immunoassay systems through the solid-phase chemiluminescent immunometric assay (CLIA) method. All kits and instruments were supplied by Siemens Healthineers GmbH, Germany. Normal assay ranges were as follows: IL-1β, 5–1000 pg/ml; IL-6, 2–1000 pg/ml; CXCL8, 5–7500 pg/ml; IL-10, 5–1000 pg/ml; TNFα, 4–1000 pg/ml; and CRP, 0.3–100 mg/l. For CRP measurements, samples were prediluted 1 in 100 in a CRP-free protein/buffer matrix prior to assay. As a result of inherent high concentrations of IL-6 in peritoneal fluid, two dilutions (1 in 50 and 1 in 100) were used to cover the reasonable expectations of dilutional requirements, using an IL-6-free protein/buffer matrix prior to assay. All other biomarkers were measured directly in the undiluted fluid.

### IMMULITE assay performance

To evaluate precision, single donor or pooled peritoneal fluid samples were selected and prepared at different concentrations, divided into aliquots, and stored at − 20 °C until use, with a fresh aliquot used for each run. For IL-1β, IL-6 and IL-10, repeatability/within-lab precision was measured by running the prepared pools once per day, for 5 days, in replicates of five. For CRP, TNFα and CXCL8, repeatability was assessed from 10 replicates run from the same sample aliquot in a single run. Linearity was assessed by diluting a peritoneal fluid pool with an analyte concentration near the upper reportable limit (or as high as the sample pool would allow), with a peritoneal fluid pool containing low concentrations of the relevant analyte to yield concentrations of 100%, 75%, 50%, 25%, 10%, 5%, 2.5%, 1%, and 0% of the original high-concentration pool. Separate pools were prepared for each analyte. All dilutions were tested in duplicate. Linearity of IMMULITE assays for peritoneal drain fluid was then analysed by weighted regression. To evaluate recovery of cytokines, peritoneal fluid samples were spiked with 2–3 solutions containing different amounts of each respective analyte. Samples for determination of recovery were prepared by making stock solutions in analyte-free protein buffer matrix. Stock serum solutions were added to peritoneal fluid samples at a ratio of 1:20. Spiked peritoneal fluid samples were assayed in duplicate, and the ratios between observed and expected concentrations were calculated.

### Data analysis

Data were analysed in R4.1 (R core team, 2017) [17] and graphed in Prism 9 (GraphPad Software, San Diego, California, USA). Biomarker thresholds were determined by maximising F1 scores across all timepoints. The F1 score is the harmonic mean between precision and recall, and is optimal for datasets with a large proportion of true negative patients. To create the 6C metric, log2 concentrations in pg/ml (CRP) or ng/ml (IL-6) were combined into an unweighted linear metric, with the boundary optimised by F1 score. Biomarker weighting was tested but found to not improve accuracy.

## Results

### Patient demographics

The study cohort comprised a total of 42 patients (22M:20F, median age 65). who underwent an anterior resection between June 2019 and June 2021. During this time period there were 66 patients who underwent an anterior resection. Recruitment was paused between March 2020 to August 2020 because of the COVID-19 pandemic. A total of 42 patients consented to take part in the study. Staffing availability limited the recruitment of patients, and only two patients declined to take part in the study. One of the 42 patients consented and planned to undergo an anterior resection and primary anastomosis; however, as a result of surgical decision-making this patient underwent a Hartmann’s procedure with no anastomosis. This patient was included in the data owing to being recruited and having consented.

Patient demographics did not differ significantly between groups (Table 1). In particular, there was no difference in operation time or the prevalence of laparoscopic versus open approach to operating between the three groups.

**Table 1:** Patient demographics and characteristics

	Anastomotic leaks (*n* = 4)	Other complications (*n* = 5)	Uncomplicated recoveries (*n* = 33)
Age in years, mean (range)	56 (31–82)	61 (54–70)	66 (40–87)
Sex			
Male	3	2	17
Female	1	3	16
Body mass index, mean (range)	32.1 (21.3–41.0)	27.1 (23.4–37.0)	27.5 (19.3–35.3)
Smoking status			
Smoker	1	0	6
Non-smoker	3	2	21
Ex	0	3	6
Chemotherapy			
Yes	1	1	3
No	3	4	30
Radiotherapy			
Yes	1	1	5
No	3	4	28
Approach			
Laparoscopic	3	4	27
Open	1	1	6
Operation time in minutes, mean (range)	285 (210–390)	225 (140–320)	250 (120–435)
Defunctioning Ileostomy			
Yes	1	4	13 (1 other^a^)
No	3	1	19
Tumour stage			
T1	0	1	4 (2 others^b^)
T2	0	2	6
T3	4	2	16
T4	0	0	5

^a^Hartmann’s procedure

^b^One patient no histological presence of tumour cells (previous radiotherapy), one patient T0 (preoperative biopsies of high-grade dysplasia suggestive of adenocarcinoma but no presence of adenocarcinoma histologically postoperatively)

There were a total of 77 samples collected from the 42 patients that were suitable for analysis and included in the results. This included 34, 34 and 9 samples from day 1, 2 and 3 respectively. Missing samples were due to three main reasons: the drains were emptied by clinical nursing staff before the research team had collected the sample; the operating surgeon requested the drain removal on day 2 postoperatively once the sample was collected; or there was too small a volume of peritoneal fluid to analyse on the IMMULITE platform.

**Fig. 1:**
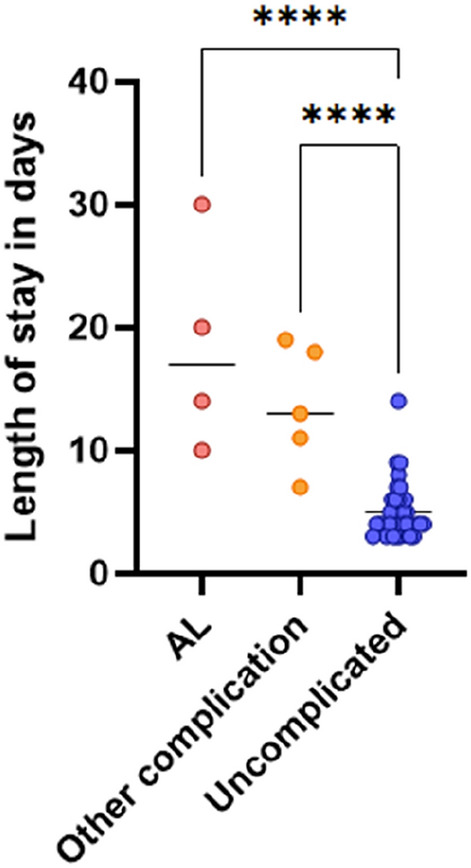
Length of hospital stay for patients undergoing colorectal surgery with uncomplicated recoveries compared to those with anastomotic leaks (AL) or other complications. Each data point indicates an individual patient; horizontal lines display the group mean. Indicated groups were significantly different by one-way analysis of variance (ANOVA) with Tukey’s post hoc test for multiple comparisons. ****p < 0.0001

Nine patients had postoperative complications; four patients had an AL and five had other complications including three patients with small bowel obstruction requiring reoperation and two patients with significant postoperative bleeds. Of the four patients who had an AL, three returned to theatre for washout with two having the anastomosis taken down and end colostomy formed, and one managed with an endosponge over several weeks. The fourth AL was managed with antibiotics alone as this patient did not become systemically unwell. Thirty-three patients had uncomplicated recoveries. Patients who experienced post-surgical complications stayed on average 7 days longer in hospital than patients with no such complications. Hospital length of stay for patients with AL was increased even further (Fig. 1), demonstrating the cost of AL to both patient health and health providers.

### Validation of drain fluid as matrix for the IMMULITE platform

The IMMULITE 1000 platform is currently only approved for plasma and serum measurements. Therefore, we first determined whether peritoneal drain fluid was a suitable substrate for the chosen measurement system. Results were found to be robust and repeatable, with coefficients of variance between 2.2% and 10.3% (Table 2) showing that local cytokine levels can be reliably determined in surgical site drain fluid using this platform. This was confirmed by assessing linearity and range of detection against ELISA-based detection, and spike-recovery profiles (Table 3). Together, these data demonstrate that the IMMULITE 1000 platform is capable of consistently determining cytokine concentrations in surgical site drain fluid to clinically relevant levels of precision.

**Table 2:** IMMULITE assay precision

IMMULITE assay	Mean concentration (pg/ml)^a^	Within-run precision (repeatability)		Within-lab precision	
		SD	CV%	SD	CV%
IL-1β	54.7	5.5	10.0	5.62	10.3
	401.4	32.3	8.1	29.78	7.4
	833.9	24.5	2.9	24.08	2.9
IL-6 (1/50 dilution)	102.9	4.6	4.5	5.02	4.9
	713.2	43.5	6.1	41.54	5.8
	1049.6	64.0	6.1	72.18	6.9
IL-6 (1/100 dilution)	86.8	5.6	6.4	6.21	7.2
	647	36.4	5.6	44.84	6.9
	808.9	42.5	5.3	51.21	6.3
IL-10	28.4	1.9	6.7	1.93	6.8
	235.2	10.4	4.4	11.68	5.0
	674.7	18.5	2.7	32.95	4.9
CXCL8	356.7	15.2	4.3	ND	ND
TNFα	206.9	4.6	2.2	ND	ND
CRP^a^	34.3	1.9	5.5	ND	ND

Within-run and within-lab precision to measure IL-1β, IL-6 and IL-10 was determined using the 5 × 5 design of a minimum of 5 test days (1 run per day) with 5 replicate measurements for each sample per run. Within-run precision to measure CRP, CXCL8 and TNFα was determined from a single sample, single run, in 10 replicates each

*SD* standard deviation, *CV* coefficient of variation, *ND* not done, *mg* milligrams, *pg* picograms

^a^CRP was measured in mg/L

**Table 3:** IMMULITE assay linearity and precision

IMMULITE assay	Linearity	Recovery (%)
IL-1β	13.35–875.7 pg/ml	92–101
IL-6 (1/50 dilution)	24.69–1002.8 pg/ml	104–110
IL-6 (1/100 dilution)	12.84–948.6 pg/ml	106–109
IL-10	28.35–666.9 pg/ml	99–107
CXCL8	31–228 pg/ml	92–109
TNFα	268–3600 pg/ml	113–114
CRP	0.1–107 mg/L	103

Linearity was analysed by weighted regression. All dilutions were tested in duplicate. To determine recoveries, spiked samples were assayed in duplicate, and observed versus expected doses were calculated

### Peritoneal immune signatures after colorectal surgery

**Fig. 2:**
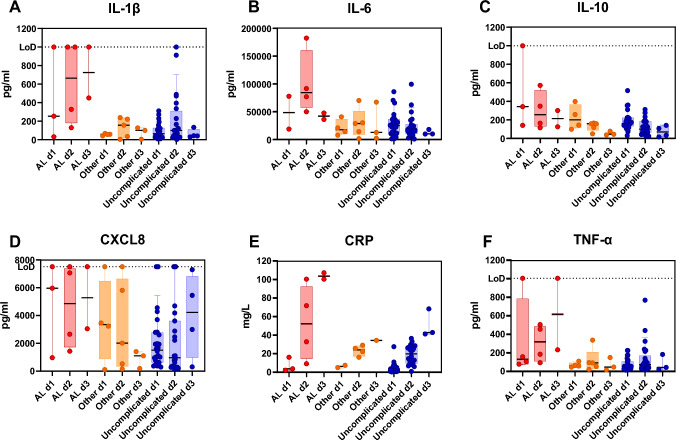
Cytokine levels in the drain fluid of patients with AL (red), other complications (orange), or uncomplicated operations (blue) over the first 3 days post-surgery. Each data point indicates an individual patient; boxes show interquartile range and whiskers extend from the 10th to 90th centiles. LoD, Limit of detection

**Fig. 3:**
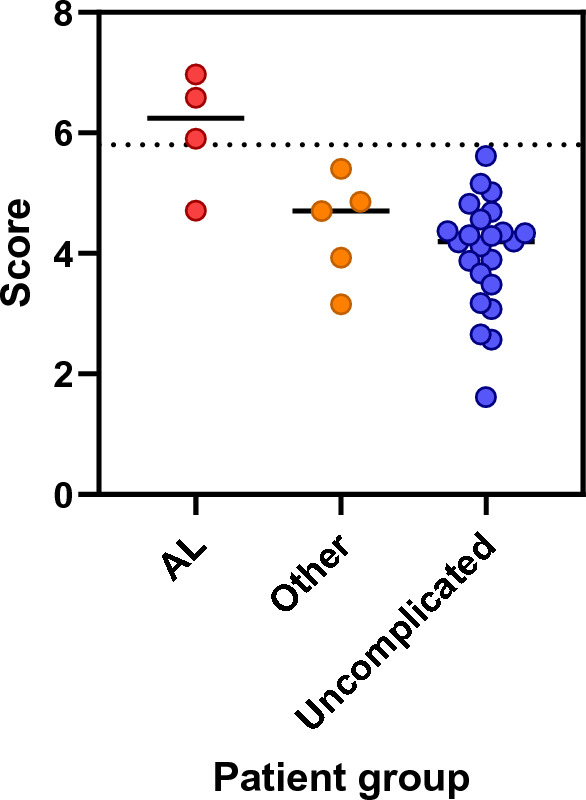
Discrimination between patients with AL and all other patients at day 2 after surgery, using an algorithm based on local levels of IL-6 and CRP. Each data point indicates an individual patient; horizontal lines show group mean. Dotted line indicates the threshold between negative and positive values

Analysis of the peritoneal drain fluid on the IMMULITE platforms demonstrated measurable amounts of the inflammatory biomarkers IL-1β, IL-6, IL-10, CXCL8, TNFα and CRP (Fig. 2). Diagnostic accuracy was high for all biomarkers, partly reflecting the fact that all chosen biomarker thresholds correctly classified 93–97% of unaffected patients (Table 4: NPV). Given that this comprises the vast majority of patients, diagnostic accuracy was correspondingly high across the entire cohort independent of the ability to correctly identify the small number of AL cases (Table 4: PPV). F1 score is an alternative measure of accuracy which is suitable for situations in which there is an imbalance between group sizes as seen in many clinical studies. An F1 score ranges between 0 and 1, with a score of 0 indicating that at one extreme no true positive samples were successfully identified, and a score of 1 indicating optimal performance, with all positive samples being correctly identified without including any false positives. We found that amongst individual biomarkers, F1 score was highest for IL-6. This could be increased further by creating a log2-based derivative algorithm which gave equal weights to IL-6 and CRP readings (the 6C metric; Fig. 3). Indeed, the 6C metric only misclassified a single patient with AL. This is particularly noteworthy given that IL-6 and CRP would also be expected to increase in other (non-infectious) scenarios of inflammation. In addition, the 6C metric correctly classified all negative control patients as well as five patients with non-AL complications.

**Table 4:** Discrimination between patients with AL and all other patients at day 2 post operation by individual markers and derived metrics

	IL-1β	IL-6	IL-10	CXCL8	CRP	TNFα	6C metric
Sensitivity	0.75	0.75	0.50	0.50	0.50	0.50	0.75
Specificity	0.79	0.96*	0.96*	0.86	1.0*	0.93	1.0*
PPV	0.33	0.60	0.67	0.33	1.0*	0.50	1.0*
NPV	0.96*	0.96*	0.93	0.93	0.94	0.93	0.97*
Diagnostic accuracy	0.79	0.94	0.91	0.82	0.94	0.88	0.97*
F1 score	0.46	0.75	0.57	0.40	0.67	0.50	0.86
Significance (*p*)^a^	0.052	0.0031	0.035	0.14	0.011	0.059	0.00081*

Measures of accuracy and effectiveness are listed in the left-hand column. Threshold values for positive/negative: IL-1β, 320 pg/ml; IL-6, 75 ng/ml; IL-10, 300 pg/ml; CXCL8, 5900 pg/ml; CRP, 70 pg/ml; TNFα, 400 pg/ml; 6C combined IL-6/CRP algorithm, 5.8

*PPV* positive predictive value,* NPV* negative predictive value, *mg* milligrams, *pg* picograms

*Scores for each measure above 0.95 and optimal *p* value

^a^Fisher’s exact test

## Discussion

For patients undergoing a surgical colorectal resection, AL is a significant complication that changes the postoperative pathway, with increased short-term morbidity and mortality, and long-term reduced cancer-specific survival. Analysis of the peritoneal fluid of the patients included in this study has demonstrated that inflammatory biomarkers are both measurable on a commercial platform and could be accurate in diagnosing an AL in the early postoperative period. Whilst this study has utilised the IMMULITE platform for the drain fluid testing, the two key markers of interest are available on other automated immunoassay platforms, such as Roche Elecsys, Beckman Coulter Access, widely available and routinely used in hospital clinical laboratories. This is the first study to show that drain fluid could be utilised in these widely available immunoassays with minimal impact on overall analytical performance, hence demonstrating the analysis and interpretation of peritoneal fluid could be comparable to blood tests. Despite the samples being frozen and interpreted as a group in this study, in clinical practice the samples would not need to be frozen and could be analysed immediately as required by hospital laboratories, further adding to the clinical applicability of this study.

The use of a peritoneal drain is still a variable practice amongst colorectal surgeons and departments particularly since the widespread adoption of ERAS principles [18]. Previous studies, including a multicentre European collaborative study, have demonstrated that the placement of a peritoneal drain did not reduce the rate of AL or allow earlier detection. The use of drains was not associated with major complications, but did increase the rates of surgical site infections and increased the length of stay [19, 20]. Each study has focused on drain placement as a method of preventing ALs; however, this pilot study demonstrates that a short-term placement of a peritoneal drain (48 h) could provide accurate diagnostic capabilities, thus justifying their placement. There is further data that a peritoneal microcatheter could be used in place of a drain, which could potentially minimise the adverse effects including wound infections and impact on ERAS implementation [21].

All biomarkers tested, with the exception of CXCL8, showed a measurable increased level in peritoneal fluid in those patients with AL at day 3, in agreement with previous published studies [11, 22]. IL-6 is an inflammatory cytokine which is upregulated in response to microbial pathogens but also tissue injury. Consequently, when measured in blood it is a non-specific marker of systemic inflammation [23]. Peritoneal IL-6 has been measured before in AL studies and has been proposed as a predictive marker for the presence of AL in patients after surgery in isolation [24]. It is broadly successful, with sensitivities and specificities in line with our measurements, but is insufficiently accurate to be used in isolation. In contrast, the present study shows that it can be combined specifically with local CRP levels derived from drain fluid to create an effective metric that can detect AL within 48 h postoperatively with high positive and negative predictive values.

CRP is commonly measured in blood where it is non-specifically increased in inflammatory and infective conditions, including in postoperative patients with no complications. CRP can act as an antibacterial effector which binds surface polysaccharides, opsonising bacteria including those that would be released into the peritoneum when an AL occurs, and activating the classical complement cascade. Systemic levels of CRP can be used as a negative predictive value of AL between 3 and 5 days postoperatively [10, 25], in keeping with its responsiveness to inflammation. CRP is commonly characterised as an acute phase protein made by hepatic cells in response to IL-6 and IL-1β [26]; however, production can also be stimulated in macrophages [27]. Our detection of elevated CRP in drain fluid 48 h postoperatively supports the hypothesis that it is produced locally in response to leakage of colonic content. Importantly, the differences in drain fluid CRP levels between patients with AL and those experiencing inflammation due to other complications at this time point show that measuring local CRP has the potential to be a more specific biomarker for AL than systemic CRP levels.

Dual detection of blood levels of IL-6 and CRP has already been proposed as a combined biomarker of AL [28]. However, while both markers have excellent negative predictive values, leading to high area under the receiver operating characteristic curve (AUROC) scores, positive predictive values for AL have remained poor so far. Our current study shows that sampling of drain fluid has both greater sensitivity for AL detection than blood markers and gives reliable results 24 h earlier. The magnitude and early detection of the inflammatory response likely reflect the proximity of the fluid to the site of the inflammatory event, which is in agreement with studies that have shown IL-6 release from peritoneal membranes within 2 h of surgery [29]. Interestingly, IL-10, which we found at levels averaging 200 times lower than IL-6, was below the level of detection in the same study. The use of drain fluid rather than blood could also potentially mitigate the general nature of IL-6 and CRP being increased in response to unrelated inflammatory stimuli, such as concurrent infections and systemic conditions. This has been shown to be the case in other contexts such as meningitis, where it was found that blood IL-6 was not as specific a marker as IL-6 in the cerebrospinal fluid [15].

While promising, this is a pilot study and needs to be replicated with more samples in a larger study. Given the distribution of the readings, it is possible that in a larger cohort IL-10 or IL-1β may also prove to be useful in a combined metric with IL-6, and investigating whether these could add to the robustness of the metric will be an important next step to the research. The patient cohort measured in the present study was also limited to those undergoing an anterior resection, and results were not stratified on tumour location in relation to the peritoneal fold or the distance of tumour/anastomosis from the anal verge. Without the inclusion of all pathologies (e.g. cancer and inflammatory bowel disease) and all colonic resections, it is difficult to assess the applicability of the results to all types of anastomosis and patients. However, this is the first study to indicate a sensitive and specific marker for AL before clinical manifestation.

The impact of an earlier diagnosis of an AL is hard to predict at present, and further research is needed into the best management strategy for patients who are predicted to develop an AL. However, an accurate method to diagnose and thus the ability to intervene and reduce the need for a defunctioning ileostomy, with its associated morbidity and need for reversal, is likely to ameliorate the risk of AL-related complications before the development of systemic sepsis and improve the patient’s pathway.

## Conclusion

This pilot study shows that is possible to measure peritoneal biomarkers in surgical drain fluid and utilise a combined metric to detect AL within 48 h of an anterior resection, using instrumentation that is widely available in large hospitals. The rapid detection of AL will allow further research into early interventions, which should improve patient outcomes including reduction in length of stay, reduced mortality and morbidity and long-term cancer survival.

## Data Availability

Data archiving is not mandated but data will be made available on reasonable request.

## References

[1] McDermott FD, Heeney A, Kelly ME, Steele RJ, Carlson GL, Winter DC (2015). Systematic review of preoperative, intraoperative and postoperative risk factors for colorectal anastomotic leaks. Br J Surg.

[2] Fujita S, Teramoto T, Watanabe M, Kodaira S, Kitajima M (1993). Anastomotic leakage after colorectal cancer surgery: a risk factor for recurrence and poor prognosis. Jpn J Clin Oncol.

[3] Petersen S, Freitag M, Hellmich G, Ludwig K (1998). Anastomotic leakage: impact on local recurrence and survival in surgery of colorectal cancer. Int J Colorectal Dis.

[4] Merkel S, Wang WY, Schmidt O (2001). Locoregional recurrence in patients with anastomotic leakage after anterior resection for rectal carcinoma. Color Dis.

[5] Walker KG, Bell SW, Rickard MJFX (2004). Anastomotic leakage is predictive of diminished survival after potentially curative resection for colorectal cancer. Ann Surg.

[6] McArdle CS, McMillan DC, Hole DJ (2005). Impact of anastomotic leakage on long-term survival of patients undergoing curative resection for colorectal cancer. Br J Surg.

[7] Lin JK, Yueh TC, Chang SC (2011). The influence of fecal diversion and anastomotic leakage on survival after resection of rectal cancer. J Gastrointest Surg.

[8] Akyol AM, Mcgregor JR, Galloway DJ, Murray GD, George WD (1991). Anastomotic leaks in colorectal cancer surgery: a risk factor for recurrence?. Color Dis.

[9] Bell SW, Walker KG, Rickard MJFX (2003). Anastomotic leakage after curative anterior resection results in a higher prevalence of local recurrence. Br J Surg.

[10] Singh PP, Zeng ISL, Srinivasa S, Lemanu DP, Connolly AB, Hill AG (2014). Systematic review and meta-analysis of use of serum C-reactive protein levels to predict anastomotic leak after colorectal surgery. Br J Surg.

[11] Reeves N, Vogel I, Ghoroghi A, Ansell J, Cornish J, Torkington J (2022). Peritoneal cytokines as a predictor of colorectal anastomotic leaks on postoperative day 1: a systematic review and meta-analysis. Tech Coloproctol.

[12] Zhang J, Friberg IM, Kift-Morgan A (2017). Machine-learning algorithms define pathogen-specific local immune fingerprints in peritoneal dialysis patients with bacterial infections. Kidney Int.

[13] Goodlad C, George S, Sandoval S (2020). Measurement of innate immune response biomarkers in peritoneal dialysis effluent using a rapid diagnostic point-of-care device as a diagnostic indicator of peritonitis. Kidney Int.

[14] Gadalla AAH, Friberg IM, Kift-Morgan A (2019). Identification of clinical and urine biomarkers for uncomplicated urinary tract infection using machine learning algorithms. Sci Rep.

[15] Cuff SM, Merola JP, Twohig JP, Eberl M, Gray WP (2020). Toll-like receptor linked cytokine profiles in cerebrospinal fluid discriminate neurological infection from sterile inflammation. Brain Commun.

[16] Rahbari NN, Weitz J, Hohenberger W (2010). Definition and grading of anastomotic leakage following anterior resection of the rectum: a proposal by the International Study Group of Rectal Cancer. Surgery.

[17] R Core Team. R 2017 A language and environment for statistical computing. R Foundation for Statistical Computing. Vienna, Austria. https://www.r-project.org/.

[18] Feldman LS, Delaney CP, Ljungqvist O, Carli F (2015) The SAGES/ERAS® Society manual of enhanced recovery programs for gastrointestinal surgery. Cham: Springer.

[19] Rolph R, Duffy JM, Alagaratnam S, Ng P, Novell R (2004). Intra-abdominal drains for the prophylaxis of anastomotic leak in elective colorectal surgery. Cochrane Database Syst Rev.

[20] EuroSurg Collaborative (2022). Intraperitoneal drain: international matched, prospective cohort study on placement and outcomes. BJS.

[21] Matthiessen P, Strand I, Jansson K (2007). Is early detection of anastomotic leakage possible by intraperitoneal microdialysis and intraperitoneal cytokines after anterior resection of the rectum for cancer?. Dis Colon Rectum.

[22] Cini C, Wolthuis A, D’Hoore A (2013). Peritoneal fluid cytokines and matrix metalloproteinases as early markers of anastomotic leakage in colorectal anastomosis: a literature review and meta-analysis. Color Dis.

[23] Scheller J, Chalaris A, Schmidt-Arras D, Rose-John S (2011). The pro and anti-inflammatory properties of the cytokine interleukin-6. Biochim Biophys Acta Mol Cell Res.

[24] Qi XY, Liu MX, Xu K (2022). Peritoneal cytokines as early biomarkers of colorectal anastomotic leakage following surgery for colorectal cancer: a meta-analysis. Front Oncol.

[25] Smith SR, Pockney P, Holmes R (2018). Biomarkers and anastomotic leakage in colorectal surgery: C-reactive protein trajectory is the gold standard. ANZ J Surg.

[26] Sproston NR, Ashworth JJ (2018). Role of C-reactive protein at sites of inflammation and infection. Front Immunol.

[27] Dong Q, Wright J (1996). Expression of C-reactive protein by alveolar macrophages. J Immunol.

[28] Sua B, Milne T, Jaung R (2022). Detection of anastomotic leakage following elective colonic surgery: results of the prospective biomarkers and anastomotic leakage (BALL) study. J Surg Res.

[29] Riese J, Schoolmann S, Beyer A, Denzel C, Hohenberger W, Haupt W (2000). Production of IL-6 and MCP-1 by the human peritoneum in vivo during major abdominal surgery. Shock.

